# A girl with short stature and dysmorphism

**DOI:** 10.1186/1687-9856-2013-S1-P42

**Published:** 2013-10-03

**Authors:** SW Cheng, LK Lee, CY Lee

**Affiliations:** 1 Caritas Medical Centre, Hong Kong

## Case presentation

A 5-year-9month old Chinese girl presented with severe growth retardation, height SDS -4.17 and body weight SDS -2.37. The mid-parental height was 151.2cm (3^rd^-10^th^ centile). She was born small for gestational age with no catch-up growth. Congenital anomalies including right dysplastic kidney and large secundum atrial septal defect were present. She had delayed development with microcephaly and dysmorphism, including triangular facies, epicanthic folds, low set ears, micronagthia, and brachydactyly. Ophthalmological assessment showed astigmatism, myopia, and left exotropia requiring surgical correction. Audiology assessment showed bilateral mild conductive hearing loss. Thyroid function, morning cortisol, serum calcium and renal function tests were normal. Metabolic disease workup including lactate, plasma for amino acid, urine metabolic screen, VLCFA, transferrin isoelectrofocusing and clotting profile were normal. CT and MRI brain were normal. Serum IGF-1 at 13 years old was 62nmol/L (+ 0.5SD). Growth hormone study with glucagon showed peak growth hormone of 32mg/L. Bone age was 12 years at chronological age of 12-year-10month. Radiograph of both hands showed brachydactyly but no proximal implantation of 1^st^ digit. Genetic study show normal karyotype, but MLPA and FISH later confirmed heterozygous terminal deletion of 15q26.2 with the genetic defect compatible with IGF-1 receptor mutation. Growth hormone therapy was refused by the patient and mother. Breast development started at 12-year-4month and menarche started at 13-year-5month of age. The growth spurt was absent with peak growth velocity of only 5.5cm/year. On the latest follow-up at 13-year-9month of age, her height was 128.8cm (-4.7SDS).

## Discussion

Our patient displayed typical phenotypic features of IGF-1 receptor mutation of heterozygous deletion of 15q26.2 with severe growth retardation. The condition could potentially benefit from growth hormone treatment according to recent literatures [1]. Our report highlights the importance of investigating for genetic causes of short stature in patients with concomitant dysmorphism and growth retardation.
